# Mucosal co-delivery of ketorolac and lidocaine using polymeric wafers for dental application

**DOI:** 10.1080/10717544.2017.1413445

**Published:** 2017-12-10

**Authors:** Gina S. El-Feky, Rania Farouk Abdulmaguid, Gamal M. Zayed, Rabab Kamel

**Affiliations:** ^a^ Department of Pharmaceutical Technology, National Research Center Cairo Egypt; ^b^ Department of Pharmaceutics, Faculty of Pharmacy, October University for Modern Sciences and Arts Cairo Egypt; ^c^ Department of Oral Medicine and Periodontology, Department Faculty of Dentistry, October University for Modern Sciences and Arts Cairo Egypt; ^d^ Department of Pharmaceutics and Industrial Pharmacy, Faculty of Pharmacy, Al-Azhar University at Assiut Assiut Egypt

**Keywords:** Sodium alginate, polyvinylpyrrolidone K-25, ketorolac/lidocaine wafer, gingivectomy, visual analogue scale (VAS), wound healing index (WHI)

## Abstract

The current study aimed to investigate the effectiveness of a developed sodium alginate and polyvinylpyrrolidone K-25 (PVP K-25) polymeric wafer for the co-delivery of ketorolac and lidocaine to soft tissues for healing and pain control following gingivectomy. Nine ketorolac/lidocaine lyophilized wafers were formulated and assessed for their hydration capacity, mucoadhesion ability and *in vitro* release profile to select the optimum system for further clinical investigation. Wafer F6 containing 2:1 sodium alginate to PVP K-25 and 10% glycerol showed optimum properties and was selected for the clinical study. Twenty patients were included in the study and the ketorolac/lidocaine wafer was assessed versus a market product. Visual pain analog was evaluated daily for the first week and wound healing index was evaluated for one week, two weeks and one month following the procedure. The developed ketorolac/lidocaine polymeric wafer proved to be an effective method of reducing pain and discomfort together with enhancing wound healing following gingivectomy.

## 1. Introduction

Wound healing within the oral cavity context is an extremely complex mechanism where multiple characters may intervene, such as cell and/or tissue interrelations, growth factors and salivary components.

The history of dental dressings dates back to 1923 when Ward introduced ‘Wondrpack’ with the aim of protecting the surgical site, splinting of soft tissue and mobile teeth, immobilization of the surgical site, preventing tooth hypersensitivity and enhancing patient comfort (Ward, 1929). Wound healing involves a sequence of molecular and cellular events including inflammation, cell migration, angiogenesis, extracellular matrix synthesis, and re-epithelialization. Principally, inflammation is a protective response to eliminate the initial cause of cell injury as well as necrotic cells and tissues resulting from the original insult. The inflammatory response is terminated when the injuries stimulus is removed and the inflammatory mediators have been dissipated, catabolized, or inhibited. Thus, healing begins very early in the process of inflammation (Martin et al., 1992), therefore, it is important to explore biomaterials and dressings to promote wound healing in the shortest time possible. In 1984, a review article discussed the positive effects of periodontal dressings. Sachs, in this article, explained the benefits of dressing for minimizing the risk of postoperative complications such as wound infection and bleeding, enhancing tissue healing by preventing physical trauma during mastication and speech and inhibiting granulation tissue formation (Sachs et al., 1984). It was long believed that covering the surgical site with periodontal dressing prevents microbial infections by decreasing plaque accumulation. The possibility to reduce post-surgical pain is among the main reasons for clinicians to cover the surgical site with dressing (Ghanbari et al., 2012).

Coepack^®^ is the most common and widely used non-eugenol dressing. Supplied as two pastes or as an auto-mixing system in syringe (Carranza & Saglie, 1990). Coepack^®^ is prepared by mixing equal lengths of paste from tubes containing the accelerator and the base until the resulting paste is of uniform color. However, and in spite being a gold standard, Coepack^®^, was assessed together with other periodontal dressings for their physical and mechanical properties (Zahra & Mahdi, 2013) and it was found that the tested dressings including Coepack^®^ showed dimensional changes after completion of their setting which may lead to the distortion of the surgical area (Bhaskar et al., 1966; Gjerdet & Haugen, 1977). Regarding its adhesion properties, Coepack^®^ was found to be generally a poor bioadhesive, it is worth mentioning, that researchers highlighted the importance of this property for its role in the prevention of microbial penetration. In addition, Coepack^®,^ might not be always applied evenly to the area of the wound so that local concentrations will vary across the wound, especially if it is suppurating.

Poor bioadhesion and uneven distribution over the wound surface do not lend dressings such as Coepack^®^ to controlled or extended delivery and in turn, there becomes a growing need for novel formulations with improved physical properties and containing pharmacological agents, which take active part in a controlled wound healing process.

Wafers are formulations prepared by freeze-drying of polymeric solutions to yield solid porous structures that can easily be applied to suppurating wound surfaces (Matthews et al., 2003) and have proven potential for mucosal wound healing (Ng & Jumaat, 2014). Wafers offer multiple advantages over other wound delivery systems including tensile strength, hydration, bioadhesivity, rheological properties, resistance to compressive forces and controlled drug release characteristics, all combined critically influence the performance of formulations applied to moist surfaces (Boateng & Catanzano, 2015). Due to their porous nature and higher surface area, they as well, have a higher drug loading capacity compared to films (Boateng et al., 2010).

ALG and PVP K-25 were selected for wafer preparation for their expected inherent ability to form coherent and stable freeze-dried wafers (Matthews et al., 2005).

Many studies have been conducted to investigate the effect of nonselective non-steroidal anti-inflammatory drugs (NSAIDs) to control postoperative pain after periodontal surgery, with generally favorable results (Betancourt et al., 2004). Ketorolac tromethamine is a nonsteroidal anti-inflammatory drug with an analgesic potency comparable with morphine, but without the opiate receptor-associated side effects (DiPalma, 1991). The beneficial effects of ketorolac are probably due to its ability to block prostaglandin synthesis by preventing the conversion of arachidonic acid to the endoperoxides. Animal studies demonstrated that the analgesic activity of ketorolac results principally from a peripheral action (Buckley & Brogden, 1990).

Lidocaine is one of the local anesthetics that are most widely used in surgical dental procedures today; when coming into contact with the nerve fiber it interrupts the propagation of the nerve impulse in a lasting and reversible manner (Pipa-Vallejo & Garcia-Pola-Vallejo, 2004; Catanzano et al., 2017).

The eventual aim of this work is to use two biocompatible inexpensive polymers to formulate a highly porous mucoadhesive structure capable of delivering and maintaining an effective concentration of a combination of ketorolac and lidocaine drugs to periodontal wound surfaces.

## 2. Materials and methods

### 2.1. Materials

Sodium alginate (ALG), polyvinylpyrrolidone K-25 (PVP K-25), and lactose were purchased from Sigma (St. Louis, MO). Ketorolac tromethamine was a kind gift from PHARCO Pharmaceutical Company (Alexandria, Egypt). Lidocaine HCl, a kind gift from MISR Pharmaceutical Company (Cairo, Egypt). Other materials were of analytical grade.

### 2.2. Methods

#### 2.2.1. Preparation of wafers

Nine wafer formulae containing ketorolac tromethamine and lidocaine hydrochloride were prepared. Two different polymers were used in the preparation; ALG and PVP K-25 in three different polymeric ratios (1:1, 1:2, and 2:1). The effect of varying the concentrations of glycerol as plasticizer was tested (0, 5, and 10% of the dry weight). The calculated amount of ALG was dissolved in 30 mL distilled water under magnetic stirring and left in the refrigerator for 24 hours. Then, the required amount of PVP K-25 was added in the specified ratio with continuous stirring till it was completely dissolved and the polymeric solution was finally left in the refrigerator for another 24 hours to remove air bubbles. After which, ketorolac tromethamine (2%), lidocaine HCl (2%) and lactose (5%) have been added. Glycerol (0, 5, and 10% of the dry weight) was then added and homogenously mixed with the solutions (Table 1). A specified amount of each of the produced solutions was poured in plastic rounded molds of 1.5 cm diameter and then lyophilized over a 30 h period from 25 °C to –50 °C and then back to 25 °C with a vacuum of 20 mTorr after initially being cooled from room temperature to –80 °C over a period of 24 h.

**Table 1.** The design of the nine prepared formulae.

Formula	SA:PVP (X1)	Glycerol % (X2)	Ketorolac	Lidocaine HCl	Lactose %
F1	1:1	0	2%	2%	5
F2	1:1	5	2%	2%	5
F3	1:1	10	2%	2%	5
F4	2:1	0	2%	2%	5
F5	2:1	5	2%	2%	5
F6	2:1	10	2%	2%	5
F7	1:2	0	2%	2%	5
F8	1:2	5	2%	2%	5
F9	1:2	10	2%	2%	5

#### 2.2.2. Characterization of wafers

##### 2.2.2.1. Morphology using scanning electron microscopy (SEM)

Freeze-dried wafers were fixed in place by means of double sided copper electrical tape and gold coated. SEM images were obtained using a JEOL JXA-840A SEM (Tokyo, Japan).

##### 2.2.2.2. Hydration capacity

The hydration capacity (HC) of the prepared wafers was carried out by incubating the samples at 37 ± 0.1 °C in 25 mL of PBS solution (pH 6.8). The wafers (*n* = 4) were initially weighed and the swelling behavior observed at predetermined time intervals (Wu et al., 2009). The samples were removed, blotted off carefully between tissue papers to remove the surface-adhered liquid droplets and reweighed to constant weight. The percentage of water uptake was calculated as follows:

$$\text{Water uptake }\left(\%\right)=\frac{100\times \left(Ws-W\right)}{W}$$

where Ws is the weight of the hydrated wafer and *W* is the initial weight of wafer.

##### 2.2.2.3. Drug incorporation efficiency

Each wafer was dissolved in 20 mL of phosphate buffer saline (PBS, pH 6.8). The resulting solutions were filtered using filter paper prior to analysis on a UV spectrophotometer to detect ketorolac and lidocaine concentrations at 318 nm and 265 nm wavelengths, respectively. The UV–visible spectrum of each ketorolac and lidocaine shows that they do not interfere with each other and therefore they can be quantitatively determined in the presence of each other (Fegade et al., 2009).

Drug incorporation efficiency was expressed as drug entrapment (%) represented by the following equation:

$$\text{EE }\left(\%\right)\;=\frac{\text{Total amount of drug in the wafer}}{\text{Initial amount of drug taken for loading studies}}\times 100$$

The individual values for three replicates were determined, and their mean values were reported.

##### 2.2.2.4. Drug content uniformity analysis

Each wafer was cut into four equal sized pieces. Each piece was dissolved in 20 mL of PBS (pH 6.8). The resulting solutions were filtered using filter paper prior to analysis on a UV spectrophotometer to detect ketorolac and lidocaine concentrations at 318 nm and 265 nm wavelengths, respectively. The absorbance of each solution was measured against a blank solution, each experiment was carried out in triplicate.

##### 2.2.2.5. In vitro release test

The nine wafers were assessed for their drug release profile. Each wafer was immersed in a beaker containing 50 mL PBS of pH 6.8 ± 0.1 as dissolution medium at 37 ± 0.1 °C with a stirring speed of 150 rpm applied with a magnetic stirrer, all beakers were covered throughout the experiment. Two milliliters of the dissolution medium were sampled at pre-determined time intervals and replenished with equal amounts of fresh medium to maintain a constant volume for 8 h. The concentration of each of ketorolac and lidocaine in each sample withdrawn from the dissolution medium was measured spectrophotometrically at wavelengths of 318 nm and 265 nm, respectively. The cumulative percentage of drugs’ released over 8 h period was determined.

#### 2.2.3. Ex vivo mucoadhesion time

The mucoadhesion of wafer formulations was tested using the modified rotating cylinder method reported by Grabovac et al. (2005). A fresh chicken pouch membrane was used as model mucosa. Mucosa was inverted and threaded on a cylinder of 2 cm diameter. Each wafer was allowed to swell for 10 min in 50 mL of phosphate buffer pH 6.8 and then gently placed onto the mucosal surface. The assembly was immersed in a beaker containing 500 mL of phosphate buffer pH 6.8 at 37 °C, with 300 rpm rotation. Time required for the wafer to detach from the mucosal surface or time to its complete erosion from the mucosa was recorded as the mucoadhesion time (Patel et al., 2007).

#### 2.2.4. Clinical study

This study was conducted on 20 patients, admitted to the outpatient clinic of the Faculty of Oral and Dental Medicine, MSA University. The study protocol and patients’ consents were approved by the ethics committee, Faculty of Pharmacy, MSA University.

The age of patients ranged from 17 to 30 years, they all needed a soft tissue gingivectomy procedure in two quadrants of the maxillary anterior teeth. Patients received scalpel gingivectomies in the right quadrant of the arch needing treatment followed by Coepack application (group A), while on the left quadrant they received the same procedure but the wound was covered by the selected ketorolac/lidocaine wafer (group B).

*Inclusion criteria*. Patients enrolled into the study were those needing esthetic crown lengthening and classified as ‘type 1’ according to Ernesto esthetic Crown Lengthening classification (Ernesto, 2004).

*Exclusion criteria.* Patients excluded from the study were those:

1. with history of antibiotic therapy in the past two months,
2. with history of corticosteroid therapy in the past two months,
3. on hormonal drugs in the past two months,
4. with diabetes mellitus,
5. with aggressive periodontitis,
6. who smoke.

Patients were all free from any systemic diseases as evidenced by ‘health questionnaire’ using Cornell’s index (Kerr & Millard, 1969). They were not receiving any medication that could affect the healing of soft tissues and bones.

The wound progress after surgical procedure was assessed as follows.

##### 2.2.4.1. Pain and discomfort assessment

This was done using ‘Visual Analogue Scale’ on the evening of the surgery and for the following six days.

Patients were given visual analog scales (VAS) which consists of a 100-mm line anchored by two extremes (‘no pain’ and ‘pain as bad as it could be’) to rate their pain. The patients were also asked to rate the degree of pain on two 10 cm horizontal VAS, one for the patient’s right side and one for the left side. The left endpoint of the scale was designated as ‘no pain’ and the right endpoint designated as ‘worst pain imaginable’. Patients were instructed to place a vertical mark at the position between the two endpoints that best described their personal perception of the pain they experienced on that particular side (Jensen et al., 1986).

*Score interpretation*. To follow up the severity of postoperative pain, the patients were asked to correlate the pain to a 10-point VAS on each assessment day. The anchor words were ‘no pain at all’ equivalent to 0 and ‘the most intense pain you can imagine’ equivalent to 10. The patients were not shown the previous pain score recording. The patients were asked to score pain for left and right side. The mean and standard deviation of the VAS values were determined in each group. Data were analyzed with a paired *t* test (*p* value <.05).

##### 2.2.4.2. Wound healing assessment

This was done using ‘wound healing index’ (WHI) which was recorded after surgery and at 1 week, 2 weeks, and 4 weeks post-surgery. WHI was calculated according to the following scoring system.

Score 1: uneventful healing with no gingival edema, erythema, suppuration, patient discomfort, or flap dehiscence; Score 2: uneventful healing with slight gingival edema, erythema, patient discomfort, flap dehiscence, or any suppuration; and Score 3: poor wound healing with significant gingival edema, erythema, patient discomfort, flap dehiscence, or any suppuration (Wikesjö et al., 1992; Huang et al., 2005).

## 3. Results

### 3.1. Preparation of wafers

Various wafer formulations listed in Table 1 contained different combinations of polymers and plasticizer. Wafers could be considered a balanced design for drug delivery to wound sites as it ensures long residence time while preventing damage to newly formed tissues (Boateng et al., 2010). Lyophilization is a preferred drying method as it offers porous stable products, extends their shelf life and allows their further storage at room temperature instead of refrigeration (Bunte et al., 2010).

Lyophilized wafers are made by freeze-drying gels containing therapeutic agents that would be applied to the mucosal surfaces (Matthews et al., 2008). The wafers then return to a gel form once applied and provide sustained release of the drug (Boateng et al., 2010). The porosity of the wafer adequately governs water uptake from the wound exudates and swells to maintain a moist healing environment, prevent cellular dehydration and facilitate collagen synthesis and angiogenesis to accelerate wound healing (Elsner & Zilberman, 2010).

ALG is an anionic polysaccharide, extracted from brown algae (Phaeophyceae) or obtained by bacterial biosynthesis from *Azotobacter* and *Pseudomonas* spp. Depending on the block content, length and distribution in the polymeric chain, ALGs possess different physical, chemical and gelling properties (Pereira et al., 2013). ALG dressings are characterized by the formation of a gel due to the exchange between the ions present in the dressing and wound exudate (Thomas et al., 2000). This gel creates a moist environment that promotes healing and facilities easy removal (Boateng et al., 2008). This together with its high tissue compatibility, low toxicity and good mucoadhesive properties allows ALGs to be used as biomaterials for wafers (Saarai et al., 2011). Polymer PVP K-25 was incorporated with the ALG to modify the drug release profile and enhance its mucoadhesiveness (Hassan et al., 2010).

### 3.2. Characterization of wafers

#### 3.2.1. Morphology using scanning electron microscopy

**Figure 1.**
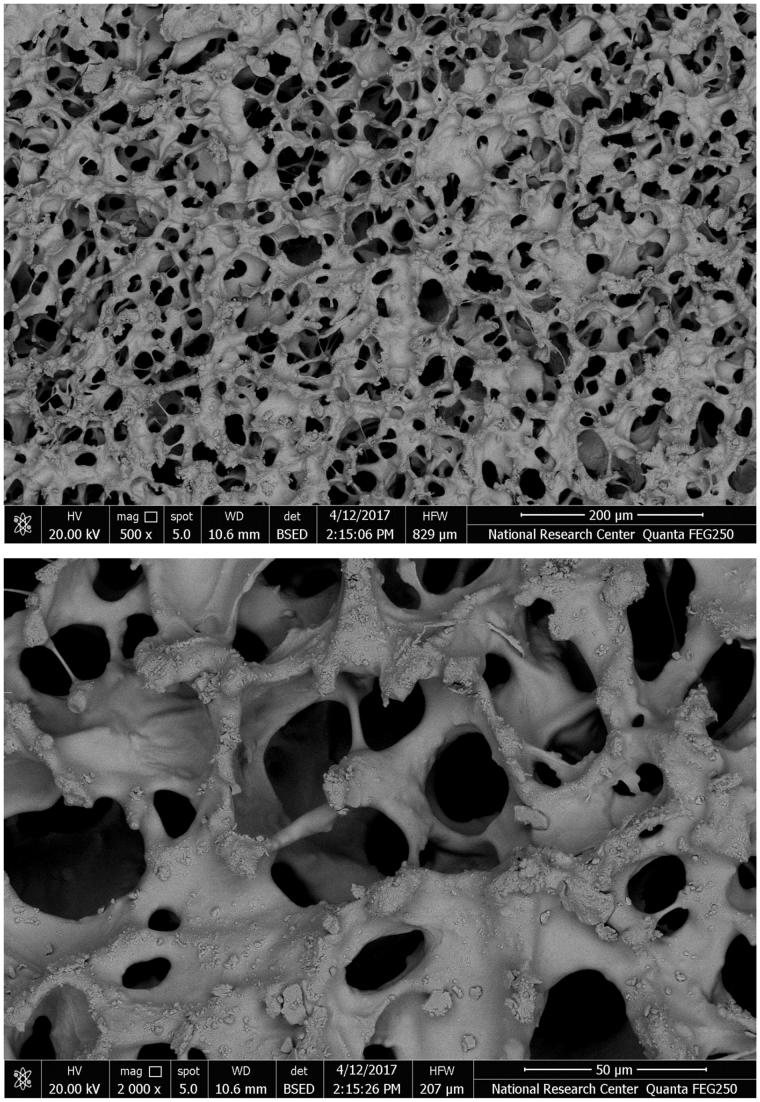
SEM micrographs of drug loaded wafers.

**Figure 2.**
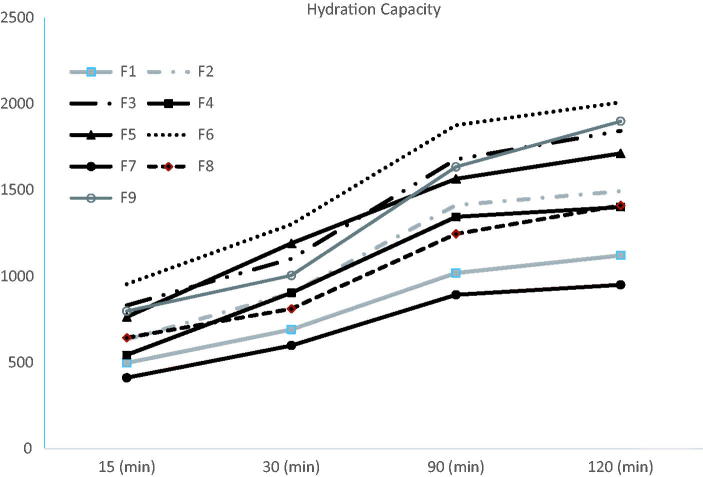
Wafers’ hydration capacity.

Scanning electron microscope results showed that the drug loaded formulations possessed an interconnecting polymeric network of sponge-like, uniform porous morphology. The average pore diameter ranged between 30 and 40 µm as shown in Figure 2. Drug particles loaded within the wafer structure appeared as crystalline depositions along the strands of the polymeric network (Figure 1).

#### 3.2.2. Hydration capacity

Figure 2 shows the HC of the nine wafers under investigation. To assess the effect of glycerol concentration on the HC, formulations F1, F2, and F3 prepared with the same SA:PVP ratio but different glycerol concentrations were compared to each other, F3 was found to possess the highest HC. The same was reported with groups of formulations F4, F5, and F6 and F7, F8, and F9. The presence of high concentrations of glycerol in wafers F3, F6, and F9 has enhanced their hydration capacities compared to their relative formulations which might be attributed to glycerol causing an increase in the intermolecular spaces between the polymer chains allowing water accessibility, this was further supported by the ‘inward-caving’ (Ayensua et al., 2012) of glycerol bearing wafers after lyophilization, this caving might have been created due to the enlarged spaces between the polymer chains by the plasticizer resulting in a loose structure capable of enhancing water absorption (Ayensua et al., 2012). However, it was seen that increasing the PVP K-25 concentration resulted in a decrease in the HC in formula F7; recording the least HC in 120 min time. Generally, at pH of 6.8 (in which the experiment was carried out), the nitrogen atom of the PVP K-25 molecule will acquire a positive charge by protonation and in turn, will cross link with SA molecules (which carry a negative charge due to ionization). Therefore, generally, increased crosslinking is expected to decrease the HC of the wafer with higher PVP K-25 concentrations (as is the case in formula F9). Whereas, formulations F6 and F3 showed insignificantly different HCs which could be a reflection of the higher concentrations of SA in such formulations; exceeding the crosslinking capacity of PVP K-25; and thus, working freely to absorb water especially in formulations with high glycerol concentration (Miranda et al., 1999; Abd El-Hady & Abd El-Rehim, 2004).

#### 3.2.3. Drug incorporation efficiency

Generally, wafers showed high drug content with no significant differences in drug content between different formulations where drug content ranged from 87.34 ± 1.60% to 93.25 ± 2.65% for ketorolac and from 86.14 ± 8.12 to 92.55 ± 11.20 for lidocaine in all tested wafers (*p*>.05, ANOVA).

#### 3.2.4. Drug content uniformity analysis

Both drugs showed uniform content distribution within each of the tested wafers (*p*>.05, ANOVA) which indicates the homogeneity of drug entrapment and subsequently, controlled drug release.

#### 3.2.5. In vitro release studies

The general high release percent of both ketorolac and lidocaine from wafers in 8 h time indicates that drug release is generally facilitated by the porous network of lyophilized wafers. The porous structure allows for an increased surface area of the dispersed drug and in turn accelerated dissolution (Bunte et al., 2010).

**Figure 3.**
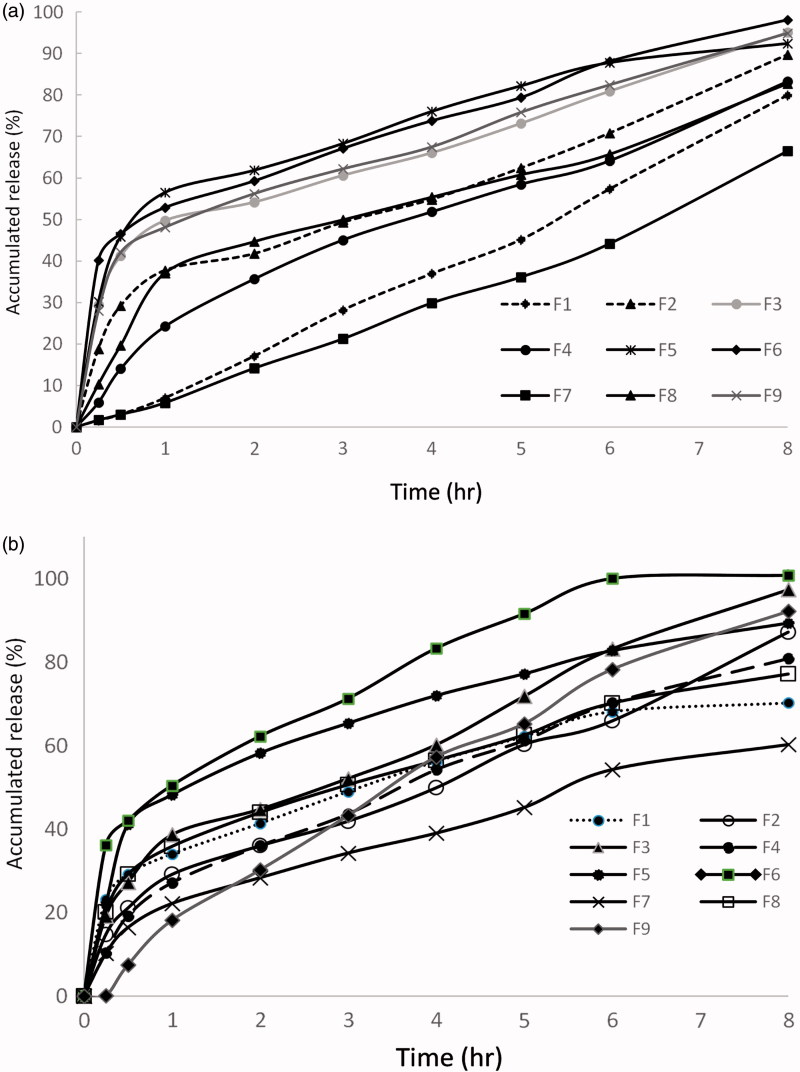
(a) Cumulative percentage release of ketorolac from the nine wafer formulations and (b) cumulative percentage release of lidocaine from the nine wafer formulations.

As shown in Figure 3, the total cumulative percent ketorolac release in 8 h from all nine wafers ranged from 66.94 ± 1.8% to 98.14 ± 3.7% for formulations F7 and F6, respectively, which was statistically significant (*p*<.05), though all formulations exhibited a sustained (controlled) release profile. In addition, the rate of release (indicated by the slope of the initial linear portion of the curve) was slower from the wafers containing 0% glycerol (F1, F4, and F7) within the first half an hour of release.

Generally, a similar trend accompanied the release of lidocaine from wafers. Lidocaine release percent ranged from 60.25 ± 2.12% from F7 to 100.76 ± 4.36% from F6.

Hydration and swelling are the main factors suggested to control the release of drugs in this study. Wafers with high ALG:PVP K-25 ratio and high glycerol percent (F6) showed faster drug release in the first hour, where, the addition of glycerol might have led to a loose and stable micro-porous structure which allowed easy hydration.

On the other hand, the low percent release of both drugs from F7 might be attributed to the combined high percentage of PVP K-25 and 0% glycerol in the formulation which could have affected the initial hydration of the wafer, thus, slowing down the rate of water access into the polymeric network and hampering to a great extent the release of drugs.

### 3.3. *Ex vivo* mucoadhesion time

The mucoadhesion of the prepared wafers at the buccal mucosa confirms their utility as buccal drug carriers since enhanced mucoadhesion results in prolonged drug residence time and thus, improved bioavailability and efficacy (Zaki et al., 2007).

It has been generally reported that the hydration of polymer is essential for the relaxation and interpenetration of polymer chains but, excess hydration generally leads to decreased mucoadhesion due to the formation of slippery mucilage (Mortazavi & Smart, 1993). As wafers uptake water, swelling starts, bonding starts and adhesion occurs. Initially, the bond formed will be weak but it is expected to increase with hydration. However, finally it reaches a point where over hydration leads to the disentanglement and distortion of polymer molecules at the interface and decreases the adhesion (Peh & Wong, 1999).

As reported in Table 2, F6 was found to have superior mucoadhesive time compared to other formulations. F3 came next whereas F7 came at the end of the list.

**Table 2.** *Ex vivo* mucoadhesive time of the nine tested drug formulations.

Formula	Mucoadhesive time (min)
F1	263
F2	299
F3	339
F4	301
F5	310
F6	364
F7	242
F8	287
F9	321

This can be attributed to the higher concentration of ALG in formula F6. ALG has free hydroxyl groups available for hydrogen binding and is an anionic polymer with carboxylate functional groups that (when ionized) can interact electrostatically with the mucin coat. Furthermore, this could also be ascribed to the conformational arrangements of d-mannuronic acid (M blocks) and l-guluronic acid (G blocks) of ALG chains that are favorable for interaction with the mucin coat, as well as the high density of carboxylate groups (Skjåk-Braek, 1992).

In spite of F9 and F7 having equal ratios of ALG:PVP K-25, it was clear that F9 had longer mucoadhesive time. This might be attributed to its high concentration of glycerin that contains large number of the hydrophilic hydroxyl groups, thus resulting in creation of a strong gel by the formation of abundance of hydrogen bonding that infiltrates intensely into the mucin layer (Mohamad et al., 2017).

### 3.4. Clinical study

Split mouth design was chosen for the present study because it minimizes the inter-subject factors such as age, sex, anatomic factors and bone metabolism and any differences that may be present (Lobo & Pol, 2015).

Based on the *in vitro* characterization results, F6 (2:1, ALG:PVP K-25 and 10% glycerol) was selected for the clinical study.

#### 3.4.1. Pain and discomfort assessment

Participants of both groups reported highest mean VAS scores (1.8 ± 0.41 and 1.7 ± 0.47 for groups A and B, respectively) on day 3. None of the participants reported mean VAS above 2. On day 6, mean VAS scores declined significantly for both groups (*p* < .05). However, comparing VAS values of both groups on day 6, a statistically significant difference in favor of group B was observed (0.7 ± 0.47 vs. 0, for groups A and B, respectively, *p* < .05).

The above findings could be explained as follows; the possibility to reduce post-surgical pain is among the main reasons for clinicians to cover the surgical site with dressing. In this respect, it has been claimed that the periodontal packs like Coepack^®^ may reduce post-operative pain and discomfort only by protecting the surgical site and they do not have therapeutic effects (Ghanbari et al., 2012). On the other hand, topical formulations of NSAIDs developed in different dosage forms such as gels, toothpastes and rinses will not only produce a local anti-inflammatory effect at the infected sites (Salvi & Lang, 2005) but can also reduce the systemic adverse effects of nonselective NSAIDs in long-term modulation of gingivitis and periodontitis-susceptible patients.

The relatively new wafer formulation of ketorolac/lidocaine offers not only a passive protection of the postsurgical wound, but, it provides a local anesthetic and anti-inflammatory drugs potentially delivered to the mucosal surface. Unlike semi solid polymer gels which flow easily after application, wafers, can maintain their swollen gel structure for a longer period and therefore, longer residence time to allow for effective treatment (Pawar et al., 2014; Catanzano et al., 2017). This might be the reason behind the slightly lower mean VAS levels on day 3 with the subsequent significant reduction on day six (reaching 0, *p* < .05) in group B.

#### 3.4.2. Wound healing assessment

Results obtained at clinical evaluation of WHI performed using both Coepack^®^ and ketorolac/lidocaine wafer showed a significant improvement in healing of the wounds from week 1 postoperative through week 4 postoperative (*p* < .05), with ketorolac/lidocaine wafer showing insignificantly lower WHI scoring vs. Coepack^®^ (1.0 vs. 1.5, respectively, *p* > .05).

**Figure 4.**
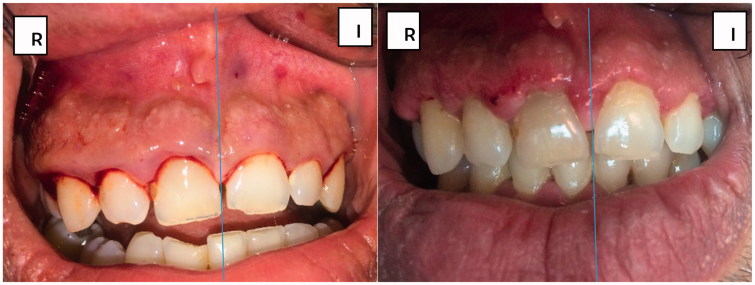
Clinical photographs taken preoperatively and after 1 week post-operatively.

In Figure 4, the patient suffered from long standing gingival inflammation, the figure is taken immediately before surgery. Figure 4(aL, aR) shows the effect of using Coepack^®^ vs. ketorolac/lidocaine wafer, respectively, on inflammation and wound healing. It is clear that swelling and inflammation are reduced with no bruising detected in Figure 4(RaR) compared to Figure 4(aL).

## 4. Conclusions

On the basis of characterization and *in vitro* release of all nine wafer formulations, formula F6 containing 2:1 ALG to PVP K-25 and 10% glycerol was the formulation of choice for the clinical study. The study was conducted on 20 patients, comparing VAS values of both groups on day 6, a statistically significant difference in favor of group B (wafer group) was reported, while clinical evaluation of WHI performed using both Coepack^®^ and ketorolac/lidocaine wafer showed a significant improvement in healing of the wounds from week 1 postoperative through week 4 postoperative (*p* < .05), with ketorolac/lidocaine wafer showing insignificantly lower WHI scoring vs. Coepack^®^. The developed ketorolac/lidocaine wafer could offer an effective method of reducing pain and discomfort and inflammation together with enhancing wound healing following gingivectomy.

## References

[CIT0001] Abd El-Hady A, Abd El-Rehim HA. (2004). Production of prednisolone by *Pseudomonas oleovorans* cells incorporated into PVP/PEO radiation cross-linked hydrogels. J Biomed Biotechnol 4:219–26. 10.1155/S1110724304308053 PMC555768 15467162

[CIT0002] Ayensua I, Mitchella JC, Boatenga JS. (2012). Development and physico-mechanical characterisation of lyophilised chitosan wafers as potential protein drug delivery systems via the buccal mucosa. Colloids Surf B: Biointerfaces 91:258–65. 22130527 10.1016/j.colsurfb.2011.11.004

[CIT0004] Betancourt JW, Kupp LI, Jasper SJ, Farooqi OW. (2004). Efficacy of ibuprofen-hydrocodone for the treatment of postoperative pain after periodontal surgery. J Periodontol 75:872–6. 15295955 10.1902/jop.2004.75.6.872

[CIT0005] Bhaskar SN, Frisch J, Margetis PM, Leonard F. (1966). Oral surgery-oral pathology conference No. 18, Walter Reed Army Medical Center. Application of a new chemical adhesive in periodontic and oral surgery. Oral Surg Oral Med Oral Pathol 22:526–35. 5330649 10.1016/0030-4220(66)90434-8

[CIT0006] Boateng J, Catanzano O. (2015). Advanced therapeutic dressings for effective wound healing—a review. J Pharm Sci 104:3653–80. 26308473 10.1002/jps.24610

[CIT0007] Boateng JS, Auffret AD, Matthews KH, et al (2010). Characterisation of lyophilised wafers and solvent evaporated films as potential drug delivery systems to mucosal surfaces. Int J Pharm 389:24–31. 20083177 10.1016/j.ijpharm.2010.01.008

[CIT0008] Boateng JS, Matthews KH, Stevens HNE, Eccleston GM. (2008). Wound healing dressings and drug delivery systems: a review. J Pharm Sci 97: 2892–923. 17963217 10.1002/jps.21210

[CIT0009] Buckley MM, Brogden RN. (1990). Ketorolac. A review of its pharmacodynamic and pharmacokinetic properties, and therapeutic potential. Drugs 39:86–109. 2178916 10.2165/00003495-199039010-00008

[CIT0010] Bunte H, Drooge DJ, Ottjes G, et al (2010). Key considerations when developing freeze-dried formulation and current trends. Pharm Technol Eur Digit 22:2–4.

[CIT0011] Carranza AC, Saglie FR. (1990). Clinical features of gingivitis In: Glickman I, Carranza FA, eds. Glickman’s clinical periodontology. 7th ed Philadelphia: Saunders, 109–25.

[CIT0012] Catanzano O, Docking R, Schofield P, Boateng J. (2017). Advanced multi-targeted composite biomaterial dressing for pain and infection control in chronic leg ulcers. Carbohydr Polym 172:40–8. 28606546 10.1016/j.carbpol.2017.05.040

[CIT0013] DiPalma JR. (1991). Ketorolac: an injectable NSAID. Am Fam Phys 43:207–10. 1986489

[CIT0014] Elsner JJ, Zilberman M. (2010). Novel antibiotic-eluting wound dressings: an in vitro study and engineering aspects in the dressing’s design. J Tissue Viabil 19:54–66. 10.1016/j.jtv.2009.11.001 19962896

[CIT0015] Ernesto AL, Dent C. (2004). Aesthetic crown lengthening: classification, biologic rationale and treatment planning consideration. Pract Proced Aesthet Dent 16:769–78. 15739921

[CIT0016] Fegade JD, Mehta HP, Chaudhari RY, Patil VR. (2009). Simultaneous spectrophotometric estimation of ofloxacin and ketorolac tromethamine in ophthalmic dosage form. Int J ChemTech Res 1:189–94.

[CIT0017] Ghanbari H, Forouzanfar A, Fatemi K, et al (2012). Modified Widman flap procedure: with or without periodontal dressing? OJST 2:170–2.

[CIT0018] Gjerdet NR, Haugen E. (1977). Dimensional changes of periodontal dressings. J Dent Res 56:1507–10. 277471 10.1177/00220345770560121701

[CIT0019] Grabovac V, Guggi D, Bernkop-Schnürch A. (2005). Comparison of the mucoadhesive properties of various polymers. Adv Drug Deliv Rev 57:1713–23. 16183163 10.1016/j.addr.2005.07.006

[CIT0020] Hassan N, Ali M, Ali J. (2010). Development and evaluation of novel buccoadhesive wafers of nimodipine for treatment of hypertension. Drug Deliv 17:59–67. 20070241 10.3109/10717540903508987

[CIT0021] Huang LH, Neiva RE, Soehren SE, et al (2005). The effect of platelet-rich plasma on the coronally advanced flap root coverage procedure: a pilot human trial. J Periodontol 76:1768–77. 16253100 10.1902/jop.2005.76.10.1768

[CIT0022] Jensen MP, Karoly P, Braver S. (1986). The measurement of clinical pain intensity: a comparison of six methods. Pain 27:117–26. 3785962 10.1016/0304-3959(86)90228-9

[CIT0023] Kerr DA, Millard HD. (1969). Oral diagnosis. 2nd ed. C.V. St. Louis: Mosby Co, 17.

[CIT0024] Lobo TM, Pol DG. (2015). Evaluation of the use of a 940 nm diode laser as an adjunct in flap surgery for treatment of chronic periodontitis. J Indian Soc Periodontol 19:43–8. 25810592 10.4103/0972-124X.145808 PMC4365156

[CIT0025] Martin P, Hopkinson-Wooley J, McClusky J. (1992). Growth factors and cutaneous wound repair. Prog Growth Factor Res 4:25–44. 1325207 10.1016/0955-2235(92)90003-z

[CIT0026] Matthews KH, Stevens HNE, Auffret AD, et al (2005). Lyophilised wafers as a drug delivery system for wound healing containing methylcellulose as a viscosity modifier. Int J Pharm 289:51–62. 15652198 10.1016/j.ijpharm.2004.10.022

[CIT0027] Matthews KH, Stevens HNE, Auffret AD, et al (2008). Formulation, stability and thermal analysis of lyophilised wound healing wafers containing an insoluble MMP-3 inhibitor and a non-ionic surfactant. Int J Pharm 356:110–20. 18280068 10.1016/j.ijpharm.2007.12.043

[CIT0028] Matthews KH, Stevens HNE, Auffret AD, et al (2003). Wafer for wounds. International Patent. Pfizer Ltd., WO 03,037,395, August 05.

[CIT0030] Miranda LF, Lugao AB, Machado LDB, Ramanathan LV. (1999). Crosslinking and degradation of PVP hydrogels as a function of dose and PVP concentration. Rad Phys Chem 55:709–12.

[CIT0031] Mohamad SA, Sarhan HA, Abdelkader H, Mansour HF. (2017). Vitamin B12e loaded buccoadhesive films as a non-invasive supplement in vitamin b12 deficiency: *in vitro* evaluation and *in vivo* comparative study with intramuscular injection. J Pharm Sci 106:1849–58. 28400198 10.1016/j.xphs.2017.03.040

[CIT0032] Mortazavi SA, Smart J. (1993). An investigation into the role of water movement and mucus gel dehydration in mucoadhesion. J Control Release 25:197–203.

[CIT0033] Ng SF, Jumaat N. (2014). Carboxymethyl cellulose wafers containing antimicrobials: a modern drug delivery system for wound infections. Eur J Pharm Sci 51:173–9. 24076463 10.1016/j.ejps.2013.09.015

[CIT0034] Patel VM, Prajapati BG, Patel MM. (2007). Effect of hydrophilic polymerson buccoadhesive eudragit patches of propranolol hydrochloride using factorial design. AAPS PharmSciTech 8:E1–8. 10.1208/pt0802045 PMC2750360 17622120

[CIT0035] Pawar HV, Boateng JS, Ayensu I, Tetteh J. (2014). Multifunctional medicated lyophilised wafer dressing for effective chronic wound healing. J Pharm Sci 103:1720–33. 24700434 10.1002/jps.23968

[CIT0036] Peh KK, Wong CF. (1999). Polymeric films as vehicle for buccal delivery: swelling, mechanical, and bioadhesive properties. J Pharm Pharm Sci 2:53–61. 10952770

[CIT0037] Pereira RA, Carvalho DC, Vaz MH, et al (2013). Development of novel alginate based hydrogel films for wound healing applications. Int J Biol Macromol 52:221–30. 23059189 10.1016/j.ijbiomac.2012.09.031

[CIT0038] Pipa-Vallejo A, Garcia-Pola-Vallejo MJ. (2004). Local anesthetics in dentistry. Med Oral Patol Oral Cir Bucal 9:440–3. 15580122

[CIT0039] Saarai A, Kasparkova V, Sedlacek T, et al (2011). Comparative study of crosslinked sodium alginate/gelatin hydrogels for wound dressing In: Mastorakis N, et al, eds. Recent researches in geography geology, energy, environment and biomedicine. Greece: WSEAS Press, 384–9.

[CIT0040] Sachs HA, Farnosh A, Checchi L, Joseph CE. (1984). Current status of periodontal dressings. J Periodontol 55:689–96. 6394736 10.1902/jop.1984.55.12.689

[CIT0041] Salvi GE, Lang NP. (2005). The effects of non-steroidal anti-inflammatory drugs (selective and non-selective) on the treatment of periodontal diseases. Curr Pharm Des 11:1757–69. 15892673 10.2174/1381612053764878

[CIT0042] Skjåk-Braek G. (1992). Alginates: biosyntheses and some structure-function relationships relevant to biomedical and biotechnological applications. Biochem Soc Trans 20:27–33. 1633956 10.1042/bst0200027

[CIT0043] Thomas A, Harding KG, Moore K. (2000). Alginates from wound dressings activate human macrophages to secrete tumour necrosis factor-alpha. Biomaterials 21:1797–802. 10905462 10.1016/s0142-9612(00)00072-7

[CIT0044] Ward AW. (1929). Postoperative care in the surgical treatment of pyorrhea. JADA 16:635–40.

[CIT0045] Wikesjö UM, Nilvéus RE, Selvig KA. (1992). Significance of early healing events on periodontal repair: a review. J Periodontol 63:158–65. 1593409 10.1902/jop.1992.63.3.158

[CIT0046] Wu ZM, Zhang XG, Zheng C, et al (2009). Disulfide cross-linked chitosan hydrogel for cell viability and controlled protein release. Eur J Pharm Sci 37:198–206. 19491006 10.1016/j.ejps.2009.01.010

[CIT0047] Zaki NM, Awad GA, Mortada ND, Abd ElHady SS. (2007). Enhanced bioavailability of metoclopramide HCl by intranasal administration of a mucoadhesive in situ gel with modulated rheological and mucociliary transport properties. Eur J Pharm Sci 32:296–307. 17920822 10.1016/j.ejps.2007.08.006

